# Biogeography of the freshwater gastropod, *Planorbella trivolvis*, in the western United States

**DOI:** 10.1371/journal.pone.0235989

**Published:** 2020-07-29

**Authors:** Kelly R. Martin, Pieter T. J. Johnson, Jay Bowerman, Jingchun Li

**Affiliations:** 1 Department of Ecology and Evolutionary Biology, University of Colorado Boulder, Boulder, Colorado, United States of America; 2 Department of Biological Sciences, University of Idaho, Moscow, Idaho, United States of America; 3 Museum of Natural History, University of Colorado Boulder, Boulder, Colorado, United States of America; 4 Bend Science Station, Bend, OR, United States of America; University of California, UNITED STATES

## Abstract

Despite the important roles of freshwater gastropods in aquatic ecosystems, the taxonomic status of many taxa is unclear, which is compounded by a lack of information on species population genetic structuring, distribution, and dispersal patterns. The objective of this study was to address the biogeography of the freshwater snail *Planorbella trivolvis* (Gastropoda: Planorbidae) in the western United States. We amplified two genetic markers (16S, COI) from individuals belonging to western USA populations and downloaded genetic data from GenBank. We utilized minimum spanning networks to assess the genetic patterns and performed Analysis of Molecular Variance and linear regression analyses to determine how geographic distance and watershed identity contributed to the observed genetic structuring. For both markers, we found that the majority of genetic variation was associated within and among populations, rather than among watersheds. Correspondingly, there was no significant effect of geographic distance on genetic distance, suggesting that long-distance dispersal was promoting gene flow between populations. The genetic similarity could reflect avian-mediated dispersal of snails along the Pacific Flyway, a major waterfowl migratory corridor. Further analysis of the population structuring across North America revealed East-West genetic structuring, suggesting that across longitudinal gradients *P. trivolvis* experiences significant genetic isolation.

## 1 Introduction

Freshwater snails play important roles in complex ecological interactions, acting as bioindicators of water quality, invasive species, and intermediate hosts for multi-host parasites [1–5]. However, freshwater mollusks also represent one of the most threatened animal groups on Earth [6, 7]. Despite the important roles freshwater gastropods play in aquatic ecology, climatology, conservation, and epidemiology, their basic biology and ecology are still understudied [2]. The taxonomic status of many taxa is uncertain, which is further impaired by a lack of information on species distribution, dispersal patterns and population structure [7]. If fundamental questions regarding freshwater gastropod ecosystem functions are to be answered, a thorough understanding of their biogeography is essential [8, 9].

One of the major factors that determines biogeographical distribution of freshwater invertebrates is their dispersal mode and ability [10, 11]. The ability to move between often discrete habitats has significant consequences for gene flow and population structuring. While many freshwater invertebrates are believed to readily disperse, analyses of their population structure show high levels of genetic differentiation [12]. Although animal vectors, wind or other mechanisms enable long-range dispersal to new water bodies, other factors may restrict gene flow among populations, including founder and priority effects [11, 12]. This discrepancy has been the focus of a larger discussion about dispersal, gene flow, and genetic differentiation in aquatic organisms [13]. Among the freshwater invertebrate taxa where information regarding their dispersal patterns and associated mechanisms remains unresolved are the freshwater gastropods.

Aquatic snails can move through the environment by both active and passive dispersal mechanisms. Active dispersal, such as crawling, is considered important at a local scale [14]. Freshwater snails mostly employ passive dispersal mechanisms for large-scale, rapid dispersal. Within water bodies, many biotic and abiotic factors, such as transport via fish and through currents, influence their passive dispersal ability [14]. For example, freshwater fishes in the Salmonidae family have been known to successfully transport several freshwater gastropod taxa including species in the families Valvatidae, Lymnaeidae and Tateidae [15–17]. Passive drifting downstream via currents is considered the most common dispersal mechanism in freshwater mollusks [14]. Freshwater snails within the families Tateidae and Hydrobiidae often drift downstream in the water column [18, 19]. However, the above mechanisms only apply to hydrologically connected water bodies. Dispersal of freshwater gastropods across non-connected habitats requires assistance from other vectors.

Extra-aquatic dispersal can occur via abiotic vectors such as flooding events or tornadoes; and biotic vectors such as mammals (including humans), insects, and birds [14]. Migratory waterfowl have long been proposed as long-distance dispersers of freshwater snails [20–26]. Gastropod taxa in the families Physidae, Planorbidae and Lymnaeidae have been found attached to feathers of the White-Faced Ibis (*Plegadis chihi*) (Vieillot) [21] and experimentally on the Tundra Swan (*Cygnus columbianus*) (Ord) [24]. This suggests that waterfowl-mediated dispersal promotes ongoing gene flow over large geographic ranges [27, 28] and could strongly influence freshwater gastropod distribution as well [29, 30].

Planorbidae is one of the most abundant and widespread freshwater gastropod families in the world [31, 32]. Among the species restricted to North America, one abundant genus is *Planorbella* (formerly *Helisoma*) [31]. Compared to other common freshwater snails, *Planorbella* is generally considered to be a less effective disperser. Species, such as *Physella acuta* (Draparnaud) (Family Physidae), are known to have higher fecundity, a shorter hatching time, and significant reproductive plasticity, which all aid stochastic dispersal [33–37]. While *Planorbella* is hermaphroditic and able to reproduce via self-fertilization, studies indicate this genus has lower viability through self-fertilization and reproduces primarily through outcrossing [38–42]. Low reproductive success through self-fertilization would reduce the probability of colonization of a new environment by a single individual [43]. In addition to lacking opportunistic life history characteristics, *Planorbella* is less effective at utilizing passive dispersal mechanisms. For instance, *Planorbella* spp. have been found attached to feathers of waterfowl, but experimental flight simulations have shown they are less likely to remain attached to a feather for any length of time [21, 24]. While it is known that other freshwater snails can be transported via the intestinal tract of birds, this method of dispersal was not found for *Planorbella* [22, 23]. Cumulatively, the results suggest that *Planorbella* is less likely to disperse long distances compared to other common freshwater snails.

Like many freshwater gastropod families, Planorbidae snails exhibit considerable diversity in shell morphology within species and share extremely homogenous anatomical traits between species [44–47], often leading to unclear species identifications [48, 49]. The variations in the diagnostic shell morphology has resulted in considerable uncertainty about *Planorbella* classification [50]. *Planorbella* was raised from a subgenus of *Helisoma* to the genus level by Burch [51], as was initially suggested by Taylor [52]. Of the previously 17 described species of *Helisoma*, 16 were moved to *Planorbella* based on differences in shell coiling, leaving only *Helisoma anceps*. However, previous work concluded that classification in *Planorbella* based on shell alone is not sufficient in distinguishing species [31]. Therefore, genetic identification based on mitochondrial markers is necessary to advance our understanding of Planorbidae taxonomy [44, 53]. Furthermore, there is a significant knowledge gap regarding the population genetic structuring of *Planorbella* in North America. To date, studies focus predominantly on Planorbidae species at global scales, with considerably less sampling within the United States [8]. Overall, a lack of information on the genetic diversity and population structuring of these species in their native range has limited opportunities to make meaningful comparisons and precluded an understanding of the family-level global genetic diversity.

The objective of the current study was to investigate the biogeographic genetic structuring of *P. trivolvis* in the western United States. Specifically, we opportunistically collected 60 samples from 22 ponds and reservoirs in the western USA and sequenced the 16S and COI mitochondrial loci. We created haplotype networks and calculated molecular diversity indexes and F_st_ values to describe levels of gene flow between the populations. An AMOVA was conducted to characterize the genetic variance within populations, among populations and across watersheds in the West Coast. By building a statistical model, we tested the relative influence of geographic distance on population genetic structuring. We further compared our findings with samples from GenBank to describe population structuring across North America. These results aim to provide insights into the genetic variation of this ecologically important group of gastropods while concurrently testing the influence of alternative hypothesized explanatory variables on population genetic structure.

## 2 Methods

### 2.1 Sampling

**Fig 1:**
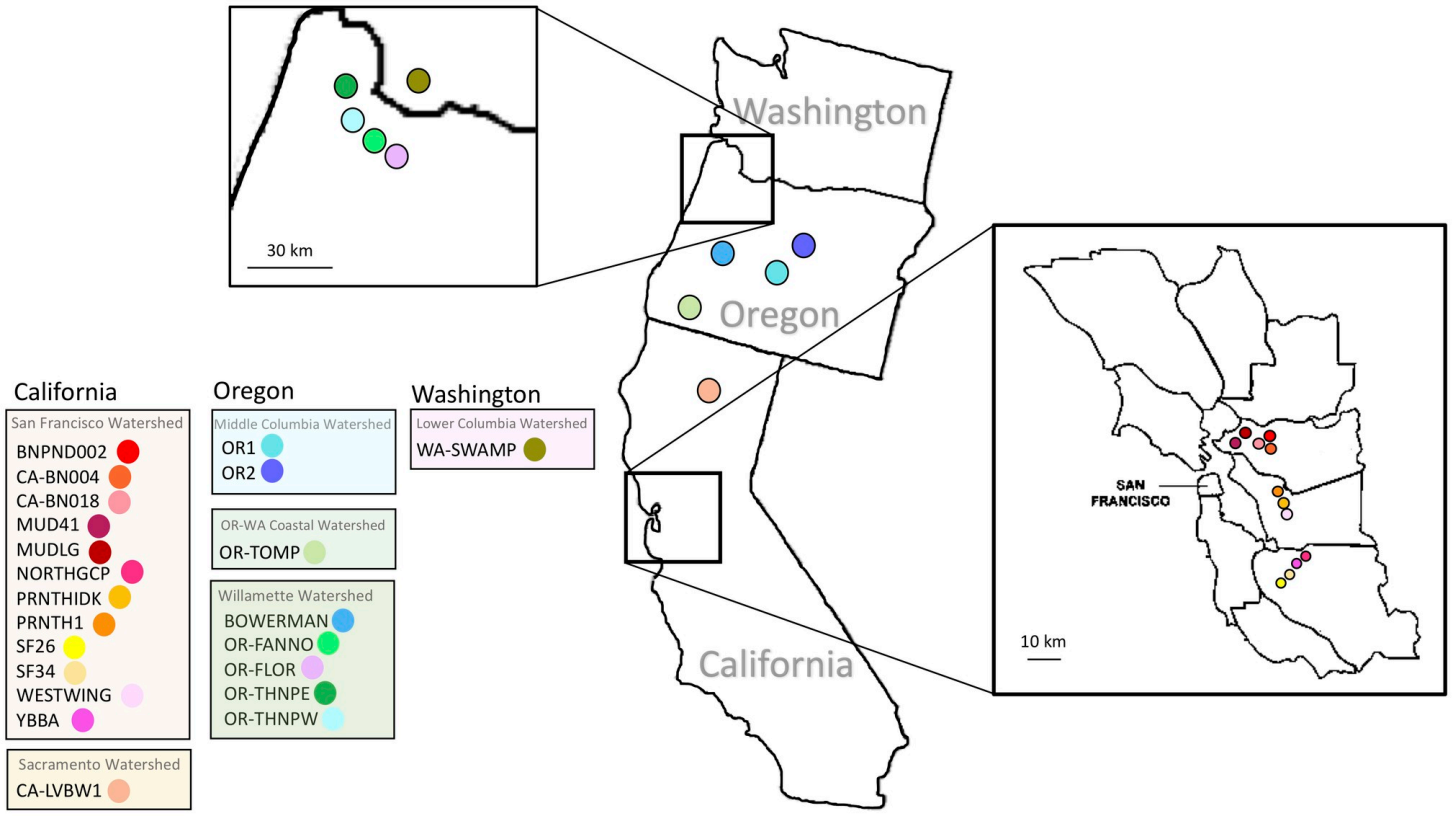
Map of the West Coast of the United States (Washington, Oregon, California) showing the 22 study sampling locations. The locality site colors correspond to each specific sampling location. The localities are grouped according to state and watershed (designated by the USGS). Detailed locality information can be found in S1 Appendix.

*Planorbella trivolvis* snails were opportunistically collected by hand or by dipnet from 22 ponds and reservoirs in California, Oregon, and Washington States (Fig 1). Samples were collected under the Scientific Take Permit (permit numbers: 029-18 and 101-19) issued by the Oregon Department of Fish and Wildlife and by the California Department of Fish and Wildlife (permit number: SC-3683). Localities included 13 sites in California, eight sites in Oregon, and one site in Washington. To assess genetic structuring, the 22 localities were grouped according to watershed identity (Fig 1), as determined using United States Geological Survey delineation maps and the 4-digit Hydrologic Unit Code (HUC) (https://water.usgs.gov/wsc/map_index.html). The localities spanned six different watersheds along the West Coast of the United States (Lower Columbia (HUC 1708), Middle Columbia (HUC 1707), Oregon-Washington Coastal (HUC 1710), Willamette (HUC 1709), Sacramento River (HUC 1802), and San Francisco Bay (HUC 1805)). Detailed locality information can be found in S1 Appendix.

At each of the 22 sites, between one and seven snails were opportunistically collected for a total of 60 specimens. The snails were identified by the collectors as *H. trivolvis*, based on diagnostic shell attributes originally described in Hubendick & Rees [54]. Both *Helisoma* and *Planorbella* are genus names used today to refer to *P. trivovlis* specimens [42, 55]. In this paper, we use the accepted name listed by the Integrated Taxonomic Information System (https://www.itis.gov/), *Planorbella trivolvis*, as described in Burch [51]. Species identities were later confirmed with genetic data (see below). Upon collection, some of the specimens were euthanized and stored in 95% EtOH. Others were kept alive, temporarily stored in the refrigerator, and subsequently transferred to 95% EtOH for DNA extraction.

### 2.2 DNA amplification

Genomic DNA was extracted from the snail foot tissue utilizing the E.Z.N.A Mollusc DNA Kit (Omega Biotek). Two mitochondrial DNA loci were amplified and sequenced (16S and COI) as both have been used successfully to recover population genetic variation in freshwater snails [9, 53, 56].

The 16S mitochondrial gene was amplified using primer sets 16Sar 5’-CGCCTGTTTATCAAAAACAT-3’ and 16Sbr 5’-CCGGTCTGAACTCAGATCACGT-3’ [57]. The COI mitochondrial gene was amplified using primer sets LCOI490 5’-GGTCAACAAATCATAAAGATATTGG-3’ and HCO2198 5’-TAAACTTCAGGGTGACCAAAAAATC-3’ [58]. PCR amplifications were performed in a total reaction volume of 12.5 *μ*L with 6.25 *μ*L GoTaq Green Master Mix (Promega), 3.75 *μ*L of nuclease-free water, 1 *μ*L of each primer and 0.5 *μ*L of the DNA template.

The PCR protocol for 16S included an initial denaturation at 94 °C for 60 s, 35 cycles of 94 °C for 30 s, 55 °C for 50 s and 72 °C for 60 s, and a final extension at 72 °C for 10 minutes [59]. The PCR protocol for COI included an initial denaturation at 96 °C for 2 minutes, 9 cycles of 96 °C for 40 s, 55 °C for 60 s and 72 °C for 60 s, 30 cycles of 96 °C for 40 s, 46 °C for 60 s and 72 °C for 60 s, and a final extension at 72 °C for 7 minutes. PCR products were assessed through gel electrophoresis.

Amplified products were sequenced by Sanger sequencing at Quintara Biosciences (California). Raw sequences were processed in CodonCode Aligner 3.1.7 to remove primer sequences and manually correct for low quality read. The final size of the 16S gene segment was 456 bp. The final size of COI gene segment was 606 bp. All *Planorbella* specimens sampled were confirmed to be *P. trivolvis* based on BLAST match with existing sequences in GenBank (>97% similarity with known *P. trivolvis* and *H. trivolvis* sequences).

In addition, all available COI sequences for *P. trivolvis* (including the one named *H. trivolvis*) were downloaded from GenBank, resulting in 38 COI sequences for *P. trivolvis*. 23 of the 38 COI sequences for *P. trivolvis* are West Coast samples and the remaining 15 are from other North American samples, including Maryland, New Mexico and Canadian localities. GenBank accession numbers and associated studies can be found in S1 Appendix.

### 2.3 Population structure

Amplified sequences were aligned using the ClustalW algorithm [60] implemented in CodonCode Aligner 3.1.7 and corrected by eye. Low quality regions at the beginning and end of the alignments were removed and not included in the final analysis. The final size of the 16S gene segment was 454 bp. The final alignment size of the COI segment was 400 bp.

**Fig 2:**
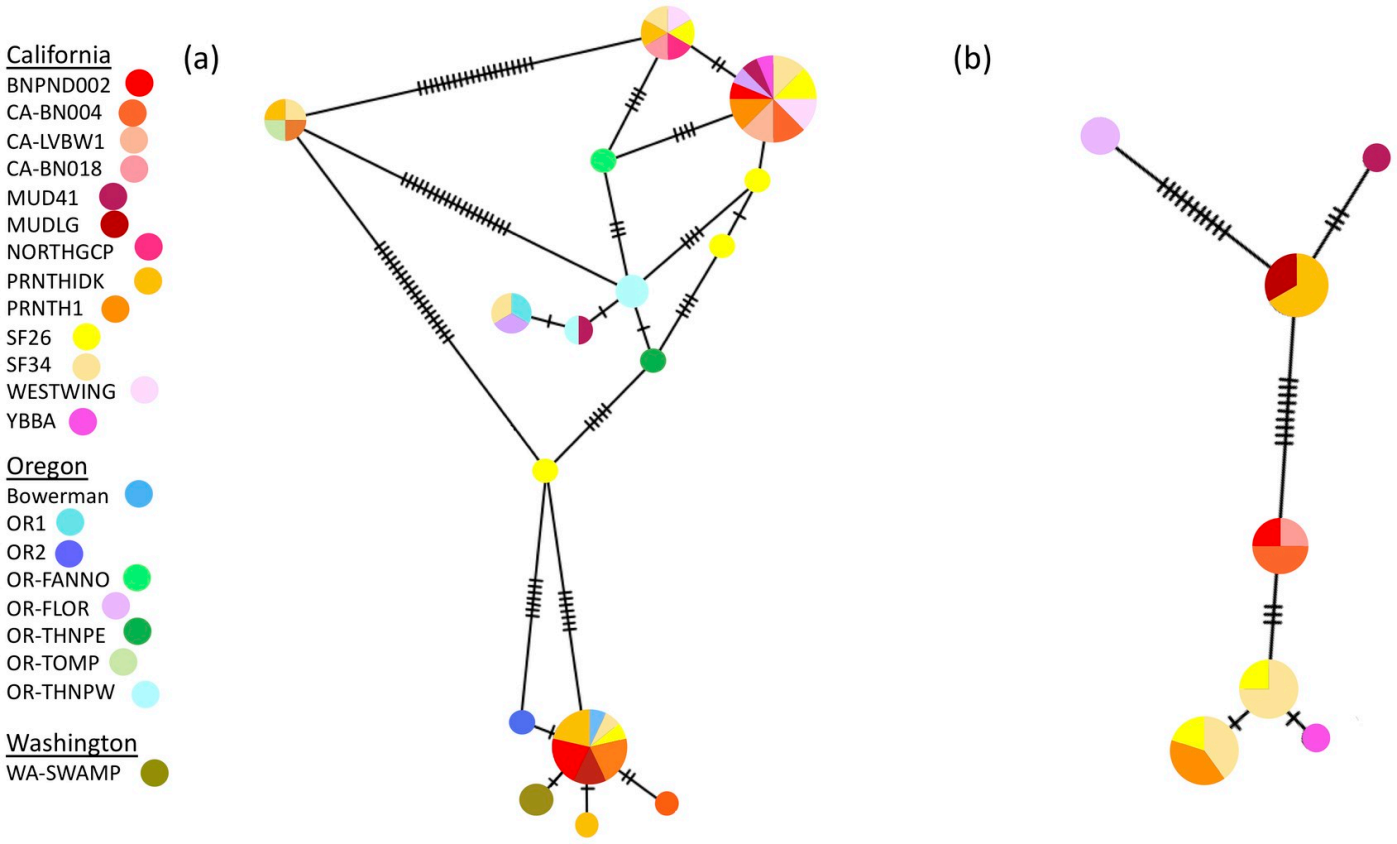
Haplotype networks for *Planorbella trivolvis* in the West Coast. (a) The 16S network of 16 haplotypes. (b) The COI network of 7 haplotypes. Each circle represents a unique haplotype. The size of the circle is proportionate to the frequency of that haplotype in the population. Each hashmark represents one base pair change. Snail haplotypes are color-coded according to their locality.

Minimum Spanning Networks (MSN) were constructed with PopARt v. 1.7 [61] (http://popart.otago.ac.nz) for both gene fragments to view the connections among haplotypes. First, we constructed MSN networks for *P. trivolvis* in their native range along the West Coast of the United States (Fig 2).

To assess the genetic structure in the West Coast, we conducted an Analysis of Molecular Variance (AMOVA) [62]. The AMOVA was performed using Arlequin v. 3.5 [63] on the 16S and COI datasets to evaluate degrees of genetic variation within populations, among populations within a watershed and among the six West Coast watersheds. Φ statistics and the variance components were calculated for within populations, among populations within watersheds, and among watersheds. Φ_ST_ estimates the genetic variation within populations; Φ_SC_ estimates the variation among populations within the watersheds; and Φ_CT_ estimates variation among all of the watersheds sampled. In order to further examine the population structure of the species, pairwise Φ_ST_ between the watersheds were calculated from the 16S and COI dataset in Arlequin v. 3.5. Significance of Φ_ST_ were determined through 10,000 random permutations (p < 0.05).

Genetic diversity summary statistics were calculated for each gene segment in Arlequin v. 3.5. Summary statistics were estimated for each watershed and across the entire sampling range. The molecular diversity indexes for each watershed included number of haplotypes (h), haplotype diversity (Hd), and nucleotide diversity. The indexes estimated for across the entire sampling range include gene diversity, mean number of pairwise differences and nucleotide diversity.

To further assess whether geographic distance is associated with *P. trivolvis* population structuring, general linear models were built using the lm function in R version 3.4.2 [64] to test for a relationship between geographic distance and genetic distance utilizing the package ggplot2. Genetic distance was the dependent variable, and geographic distance was the predictor variable. Pairwise genetic distances between West Coast localities were obtained in Arlequin v. 3.5. Very small negative values of genetic distance known to be statistical noise were rounded to 0. Geographic distance was calculated as the shortest distance between two localities on the surface of a sphere, known as the orthodromic distance.

**Fig 3:**
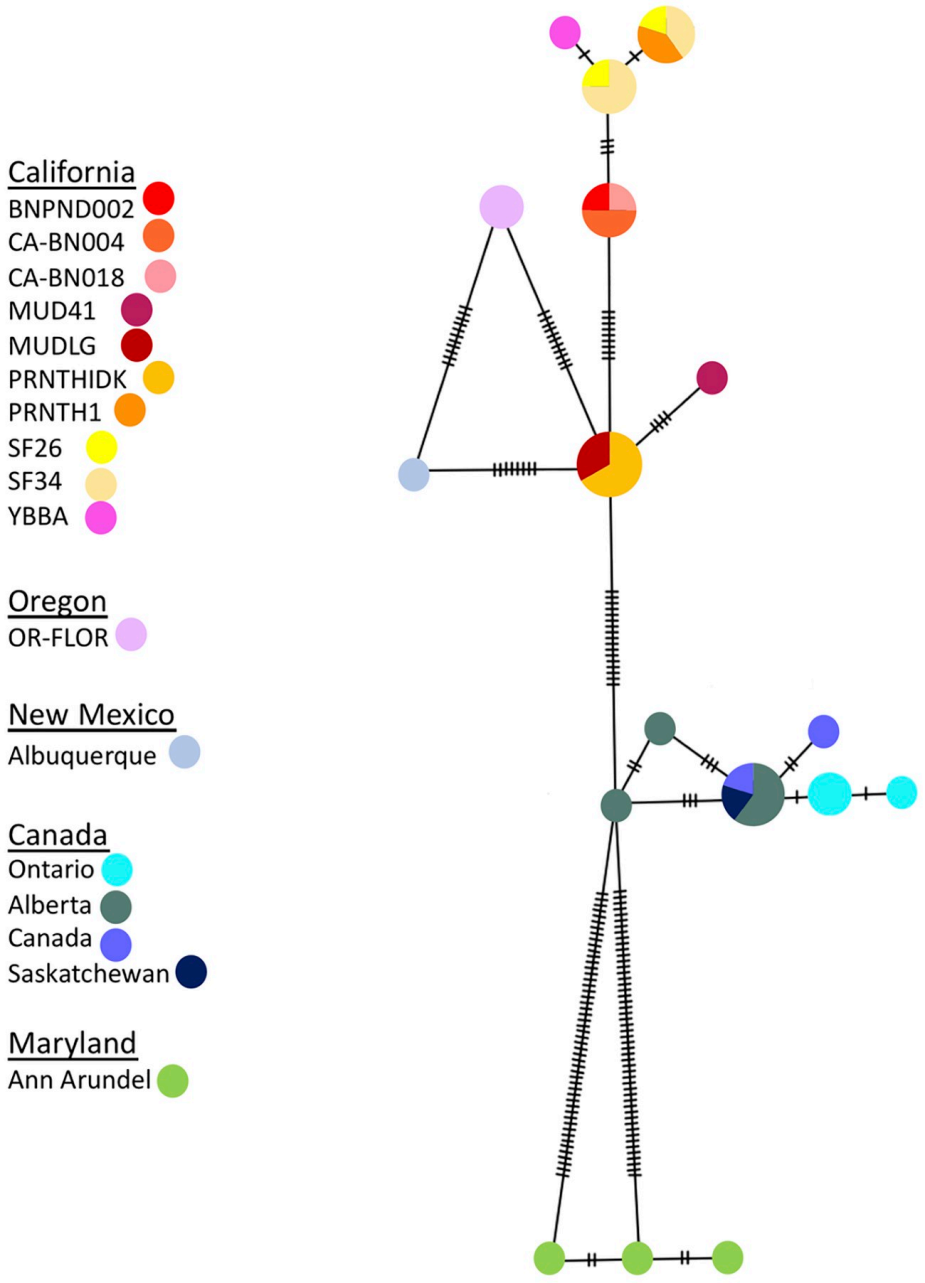
COI haplotype network for *Planorbella trivolvis* in North America. Each circle represents a unique haplotype. The size of the circle is proportionate to the frequency of that haplotype in the population. Each hashmark represents one base pair change. Snail haplotypes are color-coded according to their locality.

To assess the structuring of *P.trivolvis* across North America, we aligned the newly amplified sequences and the ones downloaded from GenBank using the ClustalW algorithm [60] implemented in CodonCode Aligner 3.1.7. Low quality regions were removed for the final analysis. We constructed a Minimum Spanning Network that included all available COI data for *P. trivolvis* (Fig 3). The average genetic distances between the sequences were calculated in MEGA X [65]. A phylogenetic reconstruction using the North American COI dataset was also conducted to compliment the network analyses. *Biomphalaria straminea* was used as an outgroup (GenBank ID KY697249, KY697248, KY697200). A maximum-likelihood reconstruction was conducted using RAxML 8.2.11 [66] with the GTR+G model and 100 bootstrap replicates. The best scoring tree is used to represent the phylogeny. Although occurrences of *P. trivolvis* have been documented globally [67], there is currently no available genetic information for us to assess *P. trivolvis* population genetics outside of North America.

## 3 Results

### 3.1 West Coast

A total of 57 out of 60 *P. trivolvis* individuals were successfully amplified for the 16S gene fragment. The genotyped individuals yielded 16 unique haplotypes, and seven singleton haplotypes are present in the populations (Fig 2). Two haplotypes were identified as the most common (H1 and H9). H1 is shared by 14 individuals from California and Oregon representing two watersheds, San Francisco and Willamette. H9 is shared by 16 individuals from California and Oregon representing three watersheds, San Francisco, Willamette and Sacramento River. Results from the AMOVA are shown in Table 1. Within population variance (Φ_ST_) accounts for 71.84% of the total genetic variation (p < 0.05), among populations within a watershed and among watershed account for 12.31% and 15.85% (p < 0.05) respectively.

**Table 1:** Analysis of Molecular Variance (AMOVA) results to test for subdivision in *Planorbella trivolvis* populations in the West Coast.

**16s (N = 57)**				
**Source of Variation**	**d.f.**	**% Variation**	**Fixation Indices**	**p-value**
Among Watershed	4	15.85	Φ_CT_ = 0.1585	**0.0301**
Among Populations within Watersheds	16	12.31	Φ_SC_ = 0.1463	0.1605
Within Populations	35	71.84	Φ_ST_ = 0.2816	**0.0111**
**COI (N = 23)**				
**Source of Variation**	**d.f.**	**% Variation**	**Fixation Indices**	**p-value**
Among Watershed	1	20.91	Φ_CT_ = 0.2091	**<0.001**
Among Populations within Watersheds	9	77.59	Φ_SC_ = 0.9811	0.0949
Within Populations	12	1.50	Φ_ST_ = 0.9850	**<0.001**

For the COI gene fragment, a total of 23 of 60 *P. trivolvis* individuals were successfully amplified. 20 of the individuals were also analyzed for the 16S gene fragment. The low amplification success for the COI gene fragment results in lower statistical power compared to the 16S gene fragment. Seven unique haplotypes were obtained as a result, and three singletons are present in the populations (Fig 2). The AMOVA suggest a significant Φ_SC_ and Φ_ST_ (p<0.001 and p<0.001, respectively). Among populations within a watershed accounts for 77.59% of the genetic variation, within population and among watershed account for 1.5% and 20.91% respectively (Table 1).

Tables 2 and 3 summarize the molecular diversity indexes for each watershed and across the entire West Coast sampling range. Haplotype diversity was high for almost every sampled watershed, even reaching 100% (Hd = 1) in some watersheds. Low haplotype diversity (Hd = 0) within certain watersheds is mostly attributed to low sample sizes. The haplotype diversity across watersheds was 0.839 for the 16S dataset and 0.85 for the COI dataset. Gene diversity for 16S was 0.8509 and for COI was 0.8498; and nucleotide diversity was 0.0414 in the 16S dataset and 0.0360 in the COI dataset.

**Table 2:** Population statistics and molecular diversity indexes for *Planorbella trivolvis* populations in the West Coast, separated by gene and watershed.

Gene	Watershed	N	H	HD	Nucleotide
**COI**	San Francisco	21	6	0.82	0.03 ± 0.02
	Willamette	2	1	0	0
	**Total**	**23**	**7**	**0.85**	
**16S**	Lower Columbia	2	1	0	0
	San Francisco	42	11	0.72	0.04 ± 0.02
	Middle Columbia	2	2	1	0.05 ± 0.05
	Willamette	8	7	0.99	0.06 ± 0.03
	Coastal	1	1	1	0
	Sacramento	2	1	0	0
	**Total**	**57**	**16**	**0.839**	

Population statistic abbreviations: N, number of individuals sampled, H, number of haplotypes, Hd, haplotype diversity, and nucleotide diversity

**Table 3:** Overall molecular diversity indexes for *Planorbella trivolvis* in the West Coast, by gene.

	COI	16S
**Gene diversity**	0.85 ± 0.04	0.85 ± 0.03
**Mean number of pairwise differences**	14.40 ± 6.70	18.79 ± 8.45
**Nucleotide diversity (average over loci)**	0.04 ± 0.02	0.04 ± 0.02

In order to further assess the population structuring of *P. trivolvis*, pairwise Φ_ST_ values were calculated between watersheds for each gene segment (Table 4). Only one marginally significant population genetic divergence was found, between the San Francisco and Willamette watersheds (p<0.05), among the six watersheds sampled. This finding supports the AMOVA results that only small amounts of variation can be attributed to the among watershed level.

**Table 4:** Population Φ_ST_ values for *Planorbella trivolvis* populations between watersheds in the West Coast, separated by gene.

Gene		1	2	3	4	5	6
**COI**	**1) San Francisco**	-	-	-	-	-	-
	**2) Willamette**	0.41	-	-	-	-	-
**16S**	**1) San Francisco**	-	-	-	-	-	-
	**2) Willamette**	**0.14**	-	-	-	-	-
	**3) Sacramento**	0.02	0.07	-	-	-	-
	**4) Lower Columbia**	0.19	0.22	1.0	-	-	-
	**5) Middle Columbia**	0.02	-0.20	0.41	0.12	-	-
	**6) Coastal**	0.54	0.46	1.0	1.0	0.45	-

Values with a p-value <0.05 are bolded.

**Fig 4:**
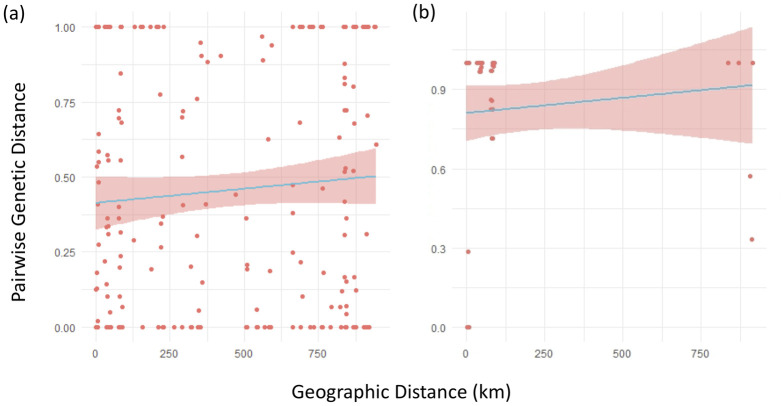
Effect of geographic distance (km) on pairwise genetic distance in *Planorbella trivolvis*. (a) The relationship between geographic distance to pairwise genetic distance in the 16S dataset. (b) The relationship between geographic distance to pairwise genetic distance in the COI dataset. Geographic distance did not determine the pairwise genetic distance (p>0.05) in either gene.

To determine whether geographic distance was associated with genetic distance, linear regressions models were produced. Within both the 16S and COI dataset, orthodromic geographic distance did not predict genetic distance, indicating there is not a significant isolation-by-distance pattern (16S: R^2^ = 0.002, p = 0.2273; COI: R^2^ = -0.006, p = 0.417) (Fig 4).

### 3.2 North America

GenBank sequences were included in this study in order to assess the overall genetic diversity of *P. trivolvis* across their entire native range. A lack of available genetic data meant we were only able to access the genetic structure of *P. trivolvis* within North America, despite its scattered global appearance [67]. Even within North America, there is only limited existing genetic data from many parts of the United States and Canada. Nonetheless, when the 15 additional GenBank COI sequences were added to create a range-wide network, the result suggested significant East-West structuring across North America (Fig 3).

**Fig 5:**
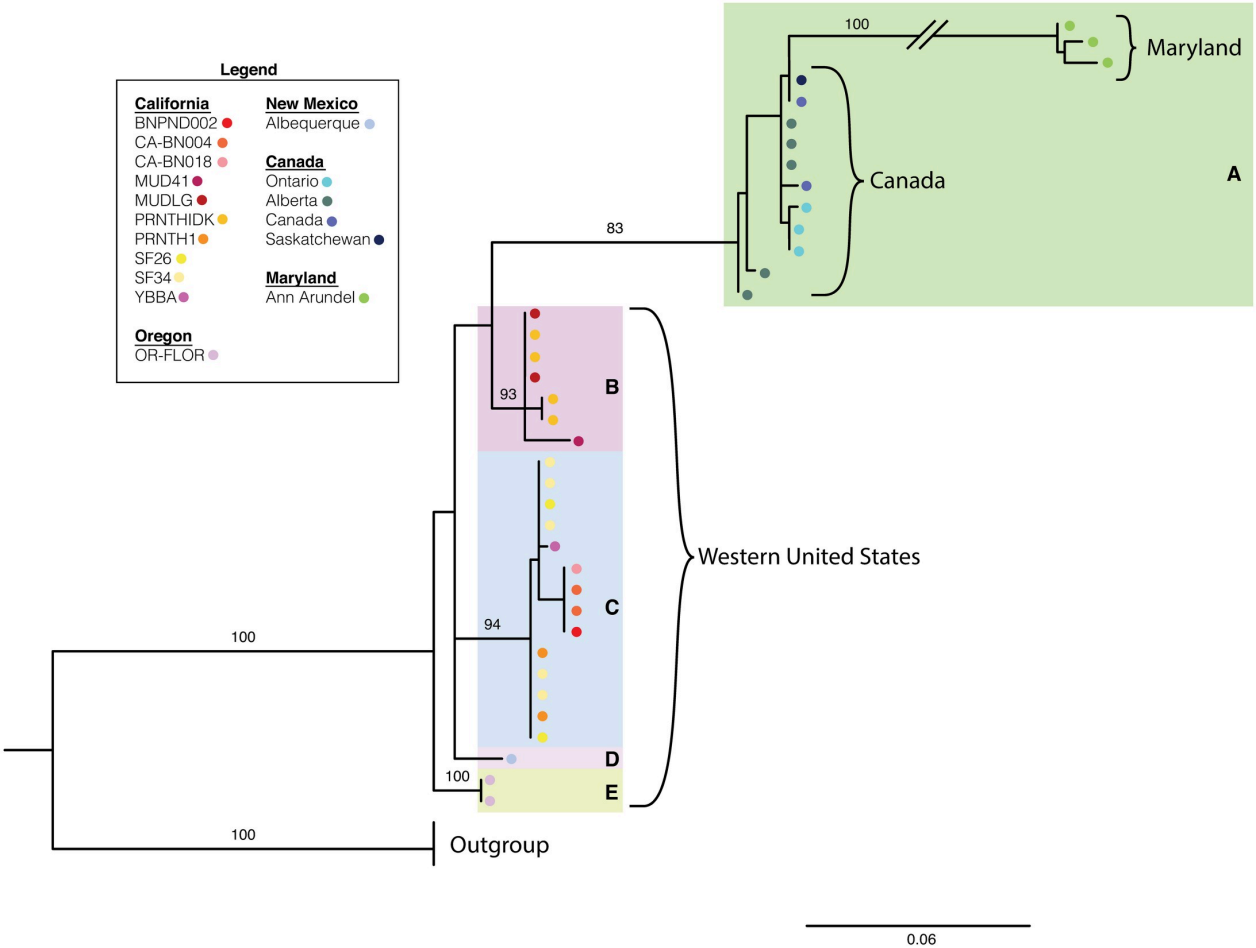
Maximum likelihood tree based on the COI dataset for *Planorbella trivolvis* samples in North America. Bootstrap values for nodes are provided when ≥83%. The tree is shaded to depict the five clades. Tips are color-coded based on sample locality.

The added GenBank samples from Canada, Maryland and New Mexico did not fall into the existing haplogroups from the West Coast but formed their own distinct clusters. The average genetic distances among the Canada haplotypes and Maryland haplotypes to the West Coast populations are 8% and 16%, respectively. The average genetic distance between the Canada haplotypes and Maryland haplotypes is 14%. These results are consistent with previous studies that show a higher similarity between freshwater organism populations in a North-South than an East-West direction in North America [27, 28]. The COI gene tree analysis of *P. trivolvis* highlights phylogenetic structuring and the presence of five well supported clades (Fig 5). Clade A includes all samples from Maryland and Canada and clades B-E include all western United States samples. Clade A contains samples as far west as Alberta, Canada where the observed East-West structuring begins. The clades largely correspond to the haplotype groups recovered in the MSN network.

## 4 Discussion

### 4.1 West Coast

The objective of this study was to address the genetic structuring of the freshwater snail *P. trivolvis* in the western United States. The results of the AMOVA provided insight into the population structuring of *P. trivolvis*. Within the 16S dataset, most of the genetic variation derived from within-population differentiation. Little variation was detected within or among watersheds, indicating overall panmixia. Within the COI dataset, most of the genetic diversity was attributed to among populations within a watershed, indicating that the species may exhibit moderate levels of population segregation within watershed. The lack of variation among watersheds is consistent with other studies examining the population structuring of freshwater gastropods, including *P. acuta* and *Taylorconcha serpenticola* (Hershler) [9, 19].

Differences in the results between the two genes could be a consequence of smaller sample sizes in the COI dataset or from the differences between the two mitochondrial genes. The COI gene used in this study is considered a universal marker because of its ability to successfully recover gene fragments and rapid rate of evolution in diverse invertebrates [58, 68, 69]. COI has been shown to evolve at a rate that is three times faster than that of 16S in certain groups [69]. The differences in the source of variation in the COI gene segment may result from the faster evolution of the COI gene that is not detected by the 16S gene. Additionally, 16S has been used to assess intrafamilial differentiation within mollusks [70]. Therefore, it may not exhibit enough variations to reveal fine scale genetic structuring compared to COI.

Another goal of this study was to determine the degree to which watershed identity (which helps reflect hydrological connectivity and passive dispersal opportunity) or geographic distance contributed to the observed genetic structuring. We found that watersheds were not a significant factor contributing to the observed population structure. Similarly, we discovered that geographic distance was unrelated to genetic distance, at least based on our measurements from 16S and COI. This result corroborates prior research, which shows that geographic distance is not strongly correlated to the genetic distance between populations of freshwater organisms [28, 71, 72]. It is noteworthy, however, in that freshwater snails generally and *Planorbella* spp. in particular are often considered to be dispersal-limited owing to the apparent dependence on hydrological connectivity for direct movement [14].

One explanation for the lack of association between watersheds, geographic distance and genetic distance is that dispersal via waterfowl is promoting gene flow across the watersheds. The Pacific Flyway, the westernmost of the four primary bird corridors in North America [73], is a major pathway for migratory birds along the Pacific Coast from Alaska to Mexico. As the waterfowl travel along this flyway, they are likely transporting the snails between sites. Indeed, avian-mediated dispersal has been identified as a potential vector for promoting long-distance gene flow in freshwater snails [21–26, 29, 30]. The occasional dispersal by waterfowl may help explain the North-South genetic similarity in this species. These findings parallel other genetically-based biogeographic assessments of freshwater gastropods which demonstrate population structuring that correlates with dispersal, specifically similar genetic patterns mapping along the Pacific Flyway [19, 74].

Importantly, a lack of geographically isolated populations suggests that despite the limited sampling of this study, most of the shared common haplotypes among populations have been recovered. If the populations were truly isolated, and thus do not share a lot of common haplotypes, the limited sampling would have retrieved a higher number of unique haplotypes. Additionally, if waterfowl were only transporting a small number of snails that successfully establish new populations, genetic bottlenecks are more likely to occur and result in more genetically diverse populations. This suggests that dispersal among populations is relatively frequent. Therefore, although a more comprehensive sampling is needed to further explore *P. trivolvi*s geography, it will likely strengthen the conclusion of this study. There is still a possibility that, by chance, we sampled more common haplotypes than what is actually present in the populations. This can also be evaluated with expanded sampling efforts in the future.

At the regional scale, watersheds are not major barriers to the distribution of *P. trivolvis*. These results contradict the historical view that *Planorbella* would be a less effective long-distance disperser when compared to other freshwater gastropods [22–24]. Their generally larger size suggests that they would be less likely to remain attached the waterfowl feathers for any length of time [24]. Remaining attached to waterfowl for long periods of time is only important if dispersal occurs over a smaller number of long-distance dispersal events. Alternatively, their migration along the Pacific Flyway may involve many small, gradual dispersal events, which is consistent with bird migration behavior along the Pacific Flyway where waterfowl utilize waterbodies as stopover points [73]. Additionally, the final size of the adult shell may not dictate their dispersal ability. First, younger snails, whether as egg-masses, newly hatched individuals or sub-adults, tend to be dispersed most often by waterfowl [75]. Second, research has shown that each species has a size at which they disperse that maximizes their success [24]. The final adult size of each species can then be considered less indicative of their dispersal ability.

Future studies are needed to examine the role of waterfowl in freshwater gastropod dispersal. The emphasis should be on smaller, more abundant birds, such as ducks and waders, that are likely important dispersers, but have received less scientific attention relative to larger birds, such as geese and swans [25]. At this time, other long-distance dispersal vectors cannot be ruled out. Anthropogenic-mediated dispersal is also unlikely to cause the observed population structuring pattern because the collection localities are isolated ponds or reservoirs not used for recreation, although the potential role of aquarium releases and the spread of invasive aquatic macrophytes—including snail “hitch-hikers”—have the potential to be influential in certain areas [76, 77].

### 4.2 North America

Results of our efforts to compile genetic sequences of *P. trivolvis* populations emphasize the degree to which this taxon is grossly under sampled for molecular analyses of population structure. Efforts to understand the population structuring are complicated by lack of available genetic data, unawareness of their global occurrence and the historic methods of identification based on morphology. This study represents the first published effort to characterize the population genetic structure of *P. trivolvis*.

The COI range-wide haplogroup network suggests that *P. trivolvis* displays strong East-West genetic structuring in their native range, despite their continuous distribution throughout North America (https://www.gbif.org/). These results imply that *P. trivolvis* experiences a high degree of genetic isolation across North America, even if such isolation was not observed along the West Coast specifically. The structuring observed in the haplotype network is further corroborated by the COI gene tree. The phylogenetic analysis supports the presence of five clades, where the Maryland and Canadian samples form their own clade (A) and exhibits long genetic distance from the western US clades B-E. The East-West genetic structuring coincides with the Rocky Mountains. Specimens collected east of the Rocky Mountains fall within clade A, and samples collected west of the Rocky Mountains fall within clades B-E. This pattern has been seen in other freshwater gastropods, such as *P. acuta* [9]. The isolation could be caused by insurmountable biogeographical barriers, a lack of dispersal vectors that traverse longitudinally across the continent, or both if the barriers also prevent dispersal of the vectors. Alternatively, the significant structuring across the United States may indicate the presence of cryptic species, as the COI genetic distances among these populations are relatively high. These genetic distances are higher than the standard 5% divergence in mtDNA between mollusk species, as seen in studies of *Rhagada* [78], *Eobania* [79], *Iberus* [80], *Helixena* [81], and *Goniobasis* species [82]. Other studies assessing the biogeographic patterns of freshwater snails have used mtDNA genetic distances of 1.3% in *Pyrgulopsis* [74] and 5% in *Physella* [9] to distinguish between species. A taxonomic study of freshwater Pleuroceridae snails found that interspecific variation in mtDNA ranged from 3.1-5.1% in *Pleurocera*, 2.2-22.6% in *Leptoxis* and up to 7.9% in *Elimia* [83]. The sequence divergences among the samples might suggest that they are distinct species. Further genetic sampling and morphological studies are needed in order to fill in the geographic gaps and determine the true taxonomic identity of *Planorbella* species in their native range.

The current global distribution of *Planorbella* is arguably unknown. Small, scattered populations of *P. trivolvis* have been identified outside North America, but their general abundance and distribution levels have yet to be elucidated [84, 85]. Despite populations of *P. trivolvis* existing outside their native range, the species is not considered invasive. One area of study relevant to their global distribution is their dispersal ability. As evidenced by its current proposed distribution, the ability of *P. trivolvis* to disperse effectively at a regional scale does not translate to the global scale. A possible explanation is that cross-continental dispersal, primarily mediated by humans, may be a less effective dispersal mechanism for *P. trivolvis*. Anthropogenic transport may occur infrequently, or the snails are not able to survive the journey. Additionally, waterfowl species that effectively disperse *P. trivolvis* in their native range may not be present in other parts of the globe, limiting *P. trivolvis* ability to successfully colonize new habitats. Alternatively, the life history traits of *Planorbella* could play a significant role in their limited global distribution. For example, once *P. trivolvis* reaches a new environment, it may be less likely to successfully establish a population. This could be a result of the species preferential outcrossing mating system, where more than just one specimen is required to start a population [43]. Ecological factors in the new environments also may prevent *P. trivolvis* from successfully establishing a population. The aquatic environments may lack suitable habitat or resources, support effective predators and competitors, or have physiochemical properties or pollution levels that *P. trivolvis* cannot tolerate [86].

The lack of knowledge about the global distribution of *Planorbella* is compounded by their phenotypic plasticity. There is a large gap in the understanding of many freshwater snails due to the traditional methods in snail identification, which rely almost exclusively on shell morphology and internal anatomy. The morphological similarity of *Planorbella* to other planorbid snail species has impeded the overall understanding of their distribution [87]. Improving their systematics will resolve current species distribution confusion and inform the study of their ecology.

A scarcity of information on the population structuring of *P. trivolvis* in their native range has prevented an understanding of their role in freshwater ecological interactions. Our results suggest that long-distance dispersal helps shape and maintain the structure of *P. trivolvis*, which can influence their ecosystem interactions both in their native and global range. Their effective dispersal has important consequences for their role as intermediate hosts to trematode parasites and conservation. Mechanical barriers, such as dams, are often proposed as a way to stop the spread of parasites in freshwater habitats by preventing the spread of their gastropod hosts [88]. Our results suggest that mechanical barriers would mitigate only local dispersal. The snails would still overcome mechanical barriers via bird-mediated dispersal; and that the parasites may also experience similar, if not more, levels of dispersal and gene flow. Additionally, this study suggests that the sustainable population growth of *P. trivolvis* relies on, in part, the frequency and abundance of contact with waterfowl. These important interactions with waterfowl depend on the availability of suitable habitat. Freshwater habitat modification threatens both aquatic and avian biodiversity by impairing the ability of freshwater snails to disperse locally and impeding contact with waterfowl that may be required for long-distance dispersal [7, 19]. Importantly, our data cannot fully address these questions and future work is necessary to understand how the dispersal patterns of *P. trivolvis* influence its ecological interactions.

## 5 Conclusions

This study addressed the population structuring of the freshwater snail *P. trivolvis* in the western United States and across North America. The observed genetic patterns were not explained by watersheds. Additionally, genetic distance was unrelated to geographic distance. The North-South genetic similarity reflects the Pacific Flyway, which suggests that long-distance dispersal vectors, such as waterfowl, are promoting ongoing gene flow over large geographic ranges and that the population structure of *P. trivolvis* may be strongly influenced by long-distance dispersal in the West Coast. Cumulatively, these results indicate that, at a regional scale, their distribution is not impeded by dispersal.

The efforts to compare the range-wide structuring of the species was complicated by lack of available information on *P. trivolvis*. This study is the first to examine the population structure of *P. trivolvis*. Our results showed that *P. trivolvis* exhibits significant East-West genetic structuring across their native range. Overall, this paper provides an initial framework for continued biogeographical analysis of *Planorbella* in their native range.

## Supporting information

S1 Appendix (CSV)
